# Permeability and Antifouling Augmentation of a Hybrid PVDF-PEG Membrane Using Nano-Magnesium Oxide as a Powerful Mediator for POME Decolorization

**DOI:** 10.3390/polym12030549

**Published:** 2020-03-03

**Authors:** Mohammed Abdulsalam, Hasfalina Che Man, Pei Sean Goh, Khairul Faezah Yunos, Zurina Zainal Abidin, Aida Isma M.I., Ahmad Fauzi Ismail

**Affiliations:** 1 Department of Biological and Agricultural Engineering, Faculty of Engineering, Universiti Putra Malaysia, UPM Serdang 43400, Selangor, Malaysia; m.abdul_22@yahoo.com; 2 Department of Agricultural and Bioresources, Ahmadu Bello University, Zaria 810107, Nigeria; 3 Advanced Membrane Technology Research Centre (AMTEC), School and Chemical and Energy Engineering, Faculty of Engineering, Universiti Teknologi Malaysia, UTM Skudai 81310, Johor, Malaysia; peisean@petroleum.utm.my (P.S.G.); afauzi@utm.my (A.F.I.); 4 Departments of Food and Process Engineering, Faculty of Engineering, Universiti Putra Malaysia, UPM Serdang 43400, Selangor, Malaysia; kfaezah@upm.edu.my; 5 Departments of Chemical and Environmental Engineering, Faculty of Engineering, Universiti Putra Malaysia, UPM Serdang 43400, Selangor, Malaysia; zurina@upm.edu.my; 6 Departments of Chemical Engineering, Segi University, Kota Damansara Selangor 47810, Malaysia; aidaisma@segi.edu.my

**Keywords:** nano-MgO, structural modification, permeability, antifouling, color rejection, POME

## Abstract

This study focused on developing a hydrophilic hybrid polyvinylidene fluoride (PVDF)-polyethylene glycol (PEG) hollow membrane by incorporating Nano-magnesium oxide (NMO) as a potent antifouling mediator. The Nano-hybrid hollow fibers with varied loading of NMO (0 g; 0.25 g; 0.50 g; 0.75 g and 1.25 g) were spun through phase inversion technique. The resultants Nano-hybrid fibers were characterized and compared based on SEM, EDX, contact angle, surface zeta-potential, permeability flux, fouling resistance and color rejection from palm oil mill effluent (POME). Noticeably, the permeability flux, fouling resistance and color rejection improved with the increase in NMO loading. PVDF-PEG with 0.50 g-NMO loading displayed an outstanding performance with 198.35 L/m^2^·h, 61.33 L/m^2^·h and 74.65% of water flux, POME flux and color rejection from POME, respectively. More so, a remarkable fouling resistance were obtained such that the flux recovery, reversible fouling percentage and irreversible fouling percentage remains relatively steady at 90.98%, 61.39% and 7.68%, respectively, even after 3 cycles of continuous filtrations for a total period of 9 h. However, at excess loading of 0.75 and 1.25 g-NMO, deterioration in the flux and fouling resistance was observed. This was due to the agglomeration of nanoparticles within the matrix structure at the excessive loading.

## 1. Introduction

Owing to the exceptional physical, mechanical and chemical stability of polyvinylidene difluoride (PVDF) polymer, its applications for bio-system/tissue engineering [1], pervaporation [2,3] and separation technology [4,5] have gained a considerable attentions. In term of solubility, PVDF polymer can easily dissolve in most of the organic solvents such as *N*,*N*-dimethylformamide (DMF), *N*,*N*-dimethylacetatamide (DMAc), Triethylphosphate (TEP) and *N*-methyl-*N*-pyrolidinone (NMP) [6,7]. As a result of this flexibility, PVDF polymer is suitable for fabricating polymeric membrane [6]. It has likewise been noted that PVDF membrane requires a relatively low pressure and minimal energy demand during filtration. This feature is most desirable for the microfiltration and ultrafiltration separation processes [8]. The aforementioned separation process using PVDF polymeric membrane have been applied on several wastewater, such as palm oil mill effluent [9], dye wastewater generated from textile industry [10], saline-water [11] and endocrine compounds [12]. Some drawbacks were reported which include continuous diminishing in permeation, low rejection as well as fouling [13]. The reduction in the membrane flux and susceptibility to fouling was collectively attributed to the hydrophobic nature of the polymer [14,15]. Thus, this justified the significant research conducted to subdue the hydrophobicity by improving the water-liking property along with permeability flux of the polymeric membrane using inorganic nanoparticles as an additive [16]. 

The commonly applied nanoparticles to modified polymeric membranes includes ZnO [17,18], TiO_2_ [9], Ag_2_O_3_ [19,20], Al_2_O_3_ [21], graphene oxide [22,23], SiO_2_ and CuO [24]. Tan et al. [17] enhanced the permeation and antifouling properties of a bared PVDF membrane by incorporating synthesized Zn-Fe oxide (ZIO) into the matrix structure. The nanocomposite membrane with 0.5 wt % ZIO loading demonstrated a significant increase in permeate flux to the magnitude of 25% increment compare to the bared PVDF membrane. Also, Shen et al. [25] modified the matrix structure of polymeric membrane using ZnO nano-additive. The result showed that the additive has sizeable influences on the pores structure as well as the hydrophilicity of the membrane. Over 254% improvement in permeability flux was reported at the loading of 0.3 g Nano-ZnO. Furthermore, the authors validated that the porosity of the modified composite membrane increased with the presence of the nano-additive, which ultimately justify the reported high flux. Nano-ZnO has large surface area that expedite the formation of hydroxyl (–OH) functional group on the surface [26,27]. The presence of –OH enhanced hydrophilicity of the PVDF polymeric membrane, thus mitigating fouling rate [26]. Analogously, TiO_2_ Ag_2_O_3_ and Al_2_O_3_ also exhibit similar features when incorporated into the matrix structure of polymeric membrane. Subramanium et al. [9] synthesized titanate nanotubes using nano-TiO_2_ as a precursor to modify the PVDF membrane. The results substantiated that under photo-catalytic condition, the involvement of TiO_2_ in the dope formulation improved the permeation consistency (35.8 L/m^2^·h) of the resultant nanocomposite membrane. Negligible fouling was observed at 0.5 wt % TNT loading throughout the 4 h continuous filtration. Another report has shown strong agreement with this observation, and the results affirmed that the antifouling performance of TiO_2_ was due to generated –OH under UV spectrum and its aptitude to exhibit self-cleaning [28]. Also, attentions are long drifted to the use of nano-Ag_2_O_3_ to modify polymeric membrane [19,20,29]. The nano-additive released Ag ions to inhibit the metabolism of the microbial-foulants, thereby preventing the generation of the extracellular polymeric substance (EPS). Thus, this ultimately curtailed the most serious type of fouling, which are organic and bio-fouling [22]. Maximous et al. [30] investigated the antifouling impact of Al_2_O_3_ and the results showed that at optimum 0.05 wt % loading, the modified composite polymeric membrane was less prone to fouling. Besides, the presence of Al_2_O_3_ in the matrix structure of a polymeric membrane not only reduces the fouling but also improves the flux consistency. 

However, most of the widely used nanoparticles, particularly as mentioned above, are photo-catalytic driven to effectively address the fouling issue [31,32]. This implies that the presence of ultraviolent radiation is a prerequisite to precede the antifouling performance. More so, the issue continues releasing antifouling radicals and superoxide could seriously jeopardize the stability of the composite matrix structure. In line with this, Tan et al. [17] reported that the structural instability of the modified nanocomposite PVDF-ZIO membrane was discernible after four filtration cycles due to the collapse of the incorporated nanoparticles. This could significantly undermine the overall antifouling performance and also re-exposing the modified membrane to inconsistencies in permeation flux along with frequent fouling challenges. Moreover, the nano- ZnO, TiO_2_, Ag_2_O_3_, Al_2_O_3_ and CuO are relatively expensive and the running cost could be unsustainable for industrial application [33,34]. Meanwhile, nano-MgO has remained one of the antimicrobial and super hydrophilic nanomaterials yet to be fully explored in improving filtration and antifouling performance of polymeric membrane [35]. The nano-MgO has the ability to release reactive oxygen species (ROS) which directly extract lipid from the cells of the microbial-foulants. This ultimately disrupted the metabolic activities and hindered biofilm formation [31,36]. More interestingly, the nano-MgO not only averts bio-fouling formation but also capable of evincing self-cleaning mechanism [31,36]. The nano-MgO precursor is readily available and comparably cheaper than other nanomaterials (such as TiO_2_, Ag_2_O_3_, Al_2_O_3_, and ZnO) [31]. Therefore, MgO-nanoparticles intimate a promising additive capable of enhancing antifouling properties and permeation performance of polymeric membranes for industrial filtration purposes. Currently, application of a modified Nano-MgO (NMO) composite PVDF-PEG membrane for separation of color pigment from POME has not been reported.

In view of these, the present study focuses on modifying the structure of an in-house fabricated PVDF-PEG ultrafiltration membrane at various loadings of NMO. The impacts of the incorporated NMO were examined based on the morphological changes, hydrophilicity, the permeability flux and fouling resistance of the resultant membrane. Furthermore, the rejection and color separation efficiency was studied, and also the used membranes were characterized using Fourier transform infrared spectroscopy. The outcome of the study demonstrated that the involvement of the NMO in the dope formulation significantly improved the antifouling properties and the permeation performance, alongside with color separation from palm oil mill effluent (POME) via the synergy of surface deprotonation and pore size screening mechanism.

## 2. Experimental Methods

### 2.1. Chemicals and Materials

Nano-MgO (NMO: particles size (BET) < 50 nm; MW = 40.30 g/Mol; purification ≥ 99.9%), Pellets PVDF (Kynar 740) and polyethylene glycol (PEG: 12,000 g/mol) were procured from Sigma Aldrich (M) Sdn Bhd, Selangor, Malaysia. The PVDF was applied as the major membrane matric polymer, while the PEG as co-polymer to enhance pore formation. The *N*,*N*-dimethylformamide (DMF: ≥99.9%; 87.12 g/mol), ethanol (≥99.98%; 46.07 g/Mol) and glycerol (≥99%; 92.09 g/Mol) were also obtained from Sigma Aldrich (M) Sdn Bhd, Selangor, Malaysia, and respectively used as doping and post-treatment solvents without any further purification. LiCl_2_.H_2_O (MW 42.39 g/Mol; ≥99%) was purchased from Acros Organic Industry, Chemicals and Reagents, Semenyih, Selangor, Malaysia and it was applied to improve the hydrophilicity of the polymers (PVDF/PEG). Also, high strength POME with initial color concentration of 8570 ADMI was collected from an Oil Palm Milling industry, Malaysia. Prior to the usage, the POME sample was filtered to remove all the visible debris and then diluted using a factor of 2 to give 4285 ADMI. This procedure was to represent the industrial final discharged color concentration ranges [9].

### 2.2. Synthesis of Nano-Hybrid PVDF/PEG-NMO

#### 2.2.1. Dope formulation

The dope formulation was preceded by adding NMO into DMF and then subjected to sonication using digital ultrasonic water bath (VWR 142-0300) at 75 °C for a period of 20 min. Subsequently, LiCl_2_·H_2_O was added into the mixture under steady agitation of 350 rpm and 75 °C for a period of 24 h. Essentially, this procedure assists in dispersion of the NMO as well as ensuring good blending of the mixtures. The mixture was followed by adding the dehydrated PVDF pellets and the co-polymer, PEG. The combination was stirred continuously at 350 rpm and 100 °C for another 24 h using a hot-plate stirrer (Monotaro; C-MAG HS7, Malaysia) to achieve a homogenous solution. The amount of the NMO contained in the dope solutions were varied as contained in Table 1.

#### 2.2.2. Spinning of Nano-Hybrid PVDF/PEG-NMO Hollow Membrane

It is important to note that same spinning parameters were applied for all of the samples. The dopes were spun through dry-jet wet swirling technique employing an annular spinneret. The inner and outside diameter of annular spinneret was 0.55 and 1.15 mm, respectively. During the fabrication of the membrane, tap water and distilled water was used as the external and internal coagulant. In addition, the pick speed control, collecting drum speed, extrusion rate, air gap, external coagulant temperature, room temperature and room humidity remains constant at 7 rpm, 11 rpm, 5 mL/min, 10 cm, 25 °C, 29.5 ± 1 °C and 72.7%, respectively. The spun fibers were drench in a continues flow water-bath at least for a period of 24 h to dislodged solvent remnants. In order to minimize shrinkage, post-treatments were applied on the fibers by immersing in ethanol for 12 h, then followed by drenching in glycerol for 5 h, respectively. The post-treated fibers were air-dried for at least 24 h to ensure complete dehydration.

### 2.3. Characterization of Nano-Hybrid PVDF/PEG-NMO Fibre

#### 2.3.1. Morphology Analysis

The cross section and surface morphology of the synthesized Nano-hybrid fiber were examined using scanning-electron-microscope, (SEM: S-3400N). Erstwhile, the composite fibers were immersed into liquid nitrogen for a period of 5 min. This procedure ensures sharp breaking of the fiber to reveal the cross sectional structure. Then, the fractured samples were sputtered coated with gold thin layer, and the voltage acceleration was maintained constant at 20 kV during the image capturing.

Also, SEM/EDX (scanning electron microscope/energy dispersive X-ray) was engaged for examining the NMO particle distribution and the dimension, as well as elemental composition in the Nano-hybrid fiber. The fractured membrane samples were place on the adhesive carbon tape of a metal plate and examined at 20 kV accelerating voltage using SEM-Thermo Scientific (Hitachi & S-3400N) for the analysis and imaging. 

#### 2.3.2. Hydrophilic and Porosity Analysis

The hydrophilicity of the synthesized membrane was analyzed based on static contact angle using goniometer (GmbH OCA 15pro, Data-Physics). The goniometer uses RO water as the probing liquid, such that the liquid droplets were captured with the equip camera after 30 s stand. The measured contact angles were analyzed using SCA20 software. In order to minimize error, each of the measurement was repeated ten times on different spots of the membrane and the average was determined as the contact angle. Throughout the analysis, the measurements were conducted at ambient temperature.

The procedure employed for the determination of porosity is based on gravimetric method [9]. Ten pieces of the synthesized membrane of 20 cm of equal length were hermetically sealed at both ends using glue-resin. The prepared samples were submersed in distilled water for 5 h under ambient temperature and humidity. Afterwards, the superficial water drops on the surface of the samples were eliminated using dry tissue paper, and then weighed as wet membrane (*M*_w_). The wet membrane samples were air dried overnight at 70 °C and reweighed as dried membrane (*M*_d_). Hence, the porosity (*ɛ*) for each sample was determined using Equation (1):

(1) $$\varepsilon ,\;\left(\%\right)=\frac{1}{\rho_{w}}\times \left(\frac{M_{w}-M_{d}}{V}\right)100$$

where ε Symbolises the membrane porosity in %, $\rho_{w}$ is density of water, ($M_{w}-M_{d}$) is the quantity of pores water in gram and *V* is the volume of the membrane sample.

#### 2.3.3. Surface Charge Analysis

The surface charge of the membranes was examined based on the potential of streaming analysis conducted using Anton SurPass Analyser (Paar Inc. Ireland). The samples were placed on the sample-holder by mean of a carbon-adhesive tape, and then the analyzer was pre-set to a maximum pressure of 400 mbar. This procedure was to ensure laminar flow throughout the analysis [17]. Also, a solution of 1 mM KCl was applied as contextual electrolyte and the pH varied between 2 and 10 was accomplished using aqueous 0.1 M HCl or NaOH. The Helmholts Smoluchowski procedure was adopted for the calculation of the Zeta-Potential surface charge.

### 2.4. Membrane Performance

#### 2.4.1. Permeability Analysis

The filtration performance was examined using a fabricated dead-end permeation system, equipped with membrane module cell and a peristaltic pump to provide the required suction pressure. Each of the modules comprises of 10 units of the membrane with equal length of 20 cm. initially, the membrane was compacted at a pressure of 0.4 MPa for a period of 30 min to ensure steady flux, while the subsequent filtration operations were performed at lower pressure of 0.3 MPa. The pure water (*J*_w_) and permeates flux (*J*_p_) were determined using Equation (2):

(2) $$J=\frac{V}{A_{s}\Delta t}$$

where *J* denotes the flux in L/m^2^·h, *V* is the volume of permeate (L), *A*_s_ is membrane surface area (m^2^) and Δt is filtration time in h.

#### 2.4.2. POME Decolorization

The membrane fibers were further subjected to filtration of diluted POME with 4285 ADMI color concentration using same set-up as applied when pure water was used as feed. Initially, the membranes fibers were engrossed in the POME solution for 90 min to initiate adhesion of thin layer color pigments on the fibers. This procedure assists in achieving accurate color rejection performances of the fibers [9]. The apparent color content of the feed POME and permeate were analyze in ADMI using UV-spectrophotometric technique (DR4000U, HACH) at an absorbance wavelength of 400 nm. The POME decolorization efficiency was determined using Equation (3):

(3) $$\%\;C_{\mathrm{removal}}=\left(1-\frac{C_{\mathrm{Permeate}}}{C_{\mathrm{Feed}\;\mathrm{POME}}}\right)\times 100$$

where, *C*_remova*l*_ is percentage of color removal in %, *C*_permeate_ is permeate color concentration in ADMI, and *C*_Feed POME_ is feed POME color concentration in ADMI.

#### 2.4.3. Fouling Analysis

In order to mimic practical situation and to evaluate the reusability, the membranes were subjected 3 cycle of continues filtration for a total period of 9 h using a known color concentration (4285 ADMI) of a diluted POME as feed. In each of the completed cycle, the antifouling performances of the membrane were evaluated after 180 min continues filtration without interruption. The indices used for the antifouling analysis were as stated in Equations (4)–(6). At every completion of each filtration cycle, the volume of POME permeates, flux and color rejected were determined. Then, the used membranes were physically cleaned under running tap-water for a period of 15 min. The washed and cleaned membranes were reapplied for the 2^nd^ cycle of POME filtration for another 180 min, following same procedure as highlighted above. In this study, the POME filtration cycle was repeated 3 times to determine the antifouling performance of the membranes using percentage of flux recovery (*FR*), reversible fouling (*RF*) and irreversible fouling (*IF*) as indices Equations (4)–(6):

(4) $$Percentage\;of\;flux\;recovery,\;\left(\%FR\right)=\frac{J_{w2}}{J_{w}}\times 100$$

(5) $$Percentage\;of\;reversible\;fouling,\;\left(\%RF\right)=\%FR-\frac{J_{P}}{J_{w}}\times 100$$

(6) Percentage of irreversible fouling, (%IF)=(1−%FR)×100

where, *J*_w2_ is the water flux after the POME filtration, L/m^2^·h.

#### 2.4.4. Characterization of used Membranes by FTIR Analysis

The surface chemical functional groups and transformation of the neat and modified membrane (0.5 g-NMO) after used were characterized using Fourier transform infrared spectroscopy (FTIR-Perkin Elmer spectrum 100 Series). The spectra analysis was taken over a wide range from 400 to 4000 cm^−1^. Essentially, the analysis involves shining a beam of light rays with variable frequencies, and then measures how much of that rays got absorbed by the specimen (i.e., the membrane samples). This ensure high signal-to-noise ratio of the spectra; thus accurate analysis of fouling level based on functional group is achievable [37]. 

## 3. Results

### 3.1. Effect of Nano-MgO on Membrane Characteristic

#### 3.1.1. Morphological Studies

**Figure 1:**
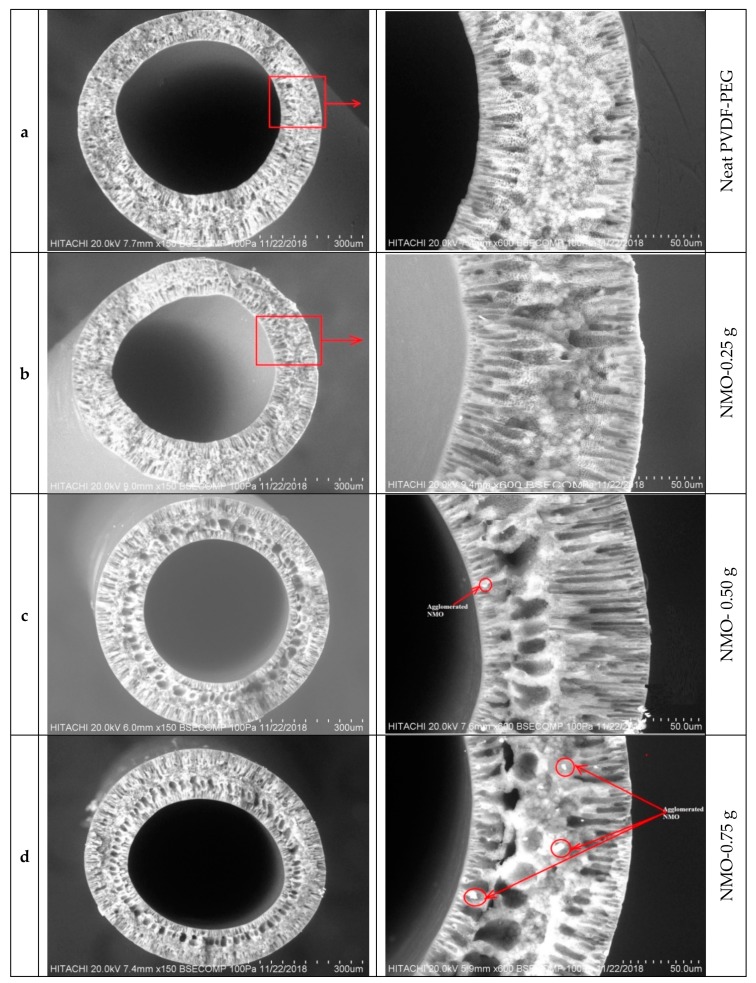

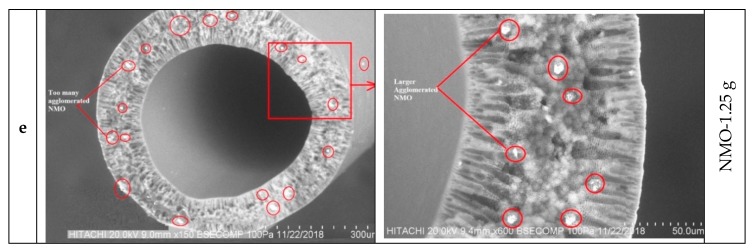
SEM Micro-structure of cross-sectional view of the Spun Fiber: (**a**–**e**) with different NMO loading.

Figure 1 presents the scanning electron microscope (SEM) images of cross-sectional view of the neat and modified hybrid Nano-MgO (NMO) PVDF-PEG membranes. It is obvious that the neat fiber had 3 distinctive layers with thin layers both at inner and outer section of the membrane. The middle layer constitutes majorly of sandwich-like morphology containing short finger-like pores at both sides toward the ultra-thin layers. However, different scenarios were observed with the modified Nanocomposite membranes. With the increasing NMO loading, the finger-like pores became longer and the number of the micro-pores structure increased considerably Figure 1b–d [38]. In addition, some spongy macrovoids were also noticed towards the inner ultra-thin layer at the higher NMO loading. This observation is in agreement with previous studies reported [38,39,40]. Though at 1.25 g-NMO loading, the pores structure was significantly suppressed and this may be due to the inhomogeneity dispersion of the Nanoparticles [25]. The uneven dispersion of the NMO at higher dosage resulted in the formation of agglomerated particles within the matrix structure [41]. Thus, the resultants membrane comprised of dense structure with suppressed pore sizes as shown in Figure 1e. From Figure 1b,c, homogenous dispersion of NMO can be observed, and this indicates a good compatibility at the loadings within the matrix structure. 

In recap, NMO loading has demonstrated a notable effect on the shape and magnitude of the pores formation. An increase in the dosage of NMO results in continuously suppression of the sandwich structure and accompanied with the increase of spongy-finger like pores at the inner and outer walls of the fiber, respectively, as indicated in Figure 1b–d. However, a contrary scenario was observed in the Figure 1e, which presented dense structure even with higher NMO loading of 1.25 g. This is indicating that a relatively uniformly distributed NMO within the polymeric matric structure were achieved at a loading range of 0.25–0.75 g to give larger surface interaction with O–H in the coagulating water bath [42]. This phenomenon often leads to formation of finger-like pore substructure and/or combination with spongy voids, fine gravimetric, porosity and thin skin layers [43]. Conversely, the tendency of non-uniformly distribution of the augmented nanoparticles (NMO) advances with increase in the dosage (1.25 g), since the spinning parameters were maintained constant for all the samples [44]. Thus, the excessive NMO got agglomerated, thereby skewing the surface interaction with O–H during crystallization. This effect in conjunction with the increase in the viscosity of the dope solution due to the higher NMO dosage, jointly delayed the demixing process, and consequently, the formation of denser-sandwich structure alongside with suppressed finger-like pore structure [25,41,43]. In addition, high NMO content dopes are in meta-stable states that are extremely supersaturated with respect to polymer crystallization [43], and as such there may exhibit a considerable amount of pre-nucleation embryos along with several aggregated Nano-particles in the dope [25,43].

**Figure 2:**
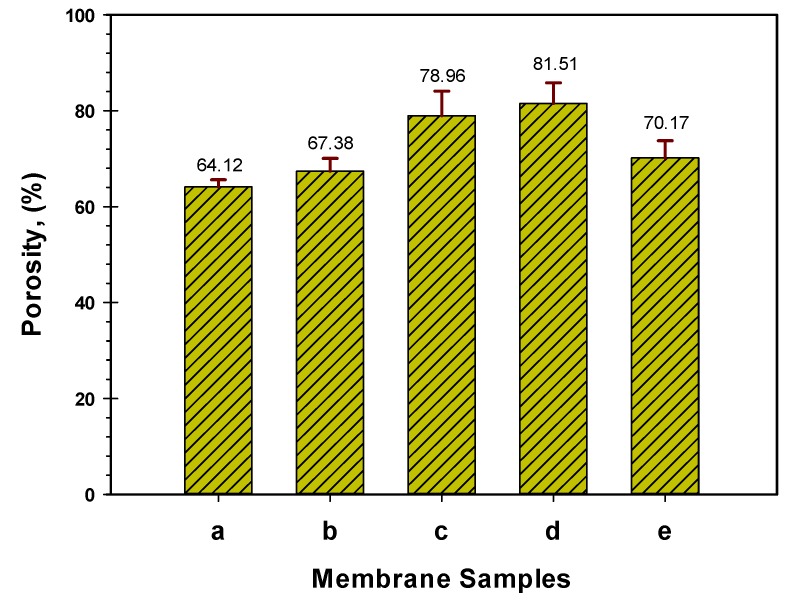
Porosity properties of neat (**a**), and modified membrane with varied NMO loading (**b**–**e**).

The results of the porosity analysis concurred with the morphological structure observed from the SEM characterization. Figure 2 displayed the porosity analysis of the spun neat and modified hollow fibers (a–e). The neat membrane (a) had the least porosity of 64.12%, while the modified membranes recorded higher values of 67.38%, 78.96%, 81.51% and 70.17% for b, c, d and e fiber, respectively. Similar remarks have been previously reported on the use of NMO to modify polymeric membranes [39,40,45]. The blended NMO accelerated the exchange speed between the non-solvent (water) and solvent (DMF) through the gelation process, thus, the porosity was increased considerably. The positive effect of the NMO on the porosity property could improve the membrane permeate flux significantly [45,46]. However, the diminished porosity percentage noticed with 1.25 g NMO loading might be due to the retardation in the crystallization which resulted from the high viscosity of the dope solution and presence of agglomerated particle at the excessive loading [47]. This effect resulted in the formation of a denser structure with suppressed pores and several visible aggregated particles, Figure 1e. Therefore, it can be deduced that the excessive NMO undermine the pores formation which could significantly diminish the porosity along with permeability of the resultant membrane.

#### 3.1.2. Membrane Hydrophilicity

**Figure 3:**
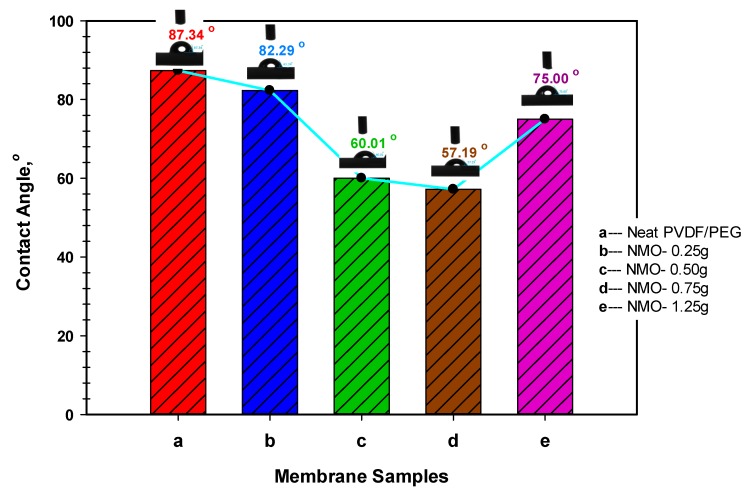
Hydrophilicity of neat PVDF-PEG (**a**), and modified Nano-hybrid membranes (**b**–**e**) with the respective goniometric images after 30 second of contact.

The hydrophilic properties of the neat and modified membranes were examined based on surface contact angle analysis. Principally, surface with a lower contact angle is said to be more hydrophilic and water-liking [48]. Results of the contact angle analysis along with the respective goniometric images for the fabricated membranes are presented in Figure 3. Essentially, the goniometric image of the dropped probing water on the horizontally-positioned membrane samples were captured after 30 s. The membrane surface hydrophilicity increased with the increasing NMO loading. As can be seen from the Figure, the neat membrane presents the highest contact angle to the magnitude of 87.34°. However, considerable reductions in the contact angle were noticed with 0.50 g-NMO and 0.75 g-NMO loading to 60.01° and 57.19°, respectively. Essentially, the decrease in the contact angles is an indication of improvements in the hydrophilicity [24]. However, at higher NMO loading of 1.25 g, the contact angle upsurges to 75° and probably this might be due to the inhomogeneity dispersion and aggregation of the NMO particles which essentially reduced its overall effect. In a whole, the hydrophilicity of the modified membrane has shown a correlation with the dosage of the NMO, which also influences the pores morphology [40], formation of hydroxyl functional group [17,39] and surface charges [42].

#### 3.1.3. Elemental Analysis

**Figure 4:**
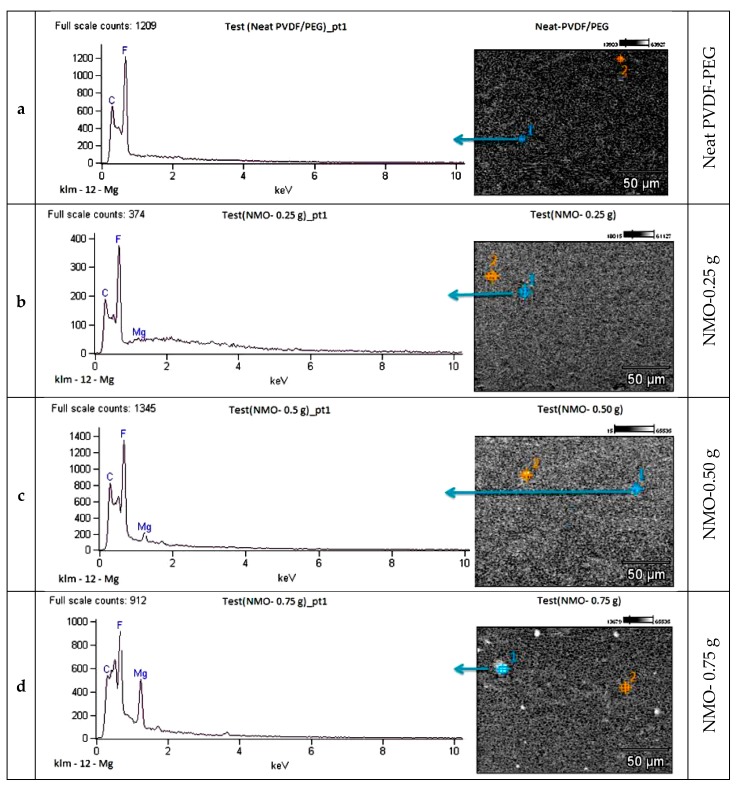

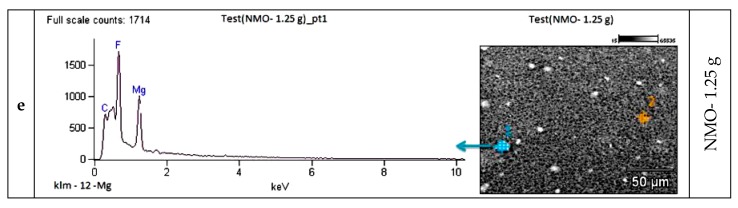
SEM/EDX spectra Mapping the Presence of NMO in (**a**–**e**) membrane.

EDX mapping was used to examine the presence of NMO within the matrix structure of the spun hollow fibers. Figure 4a presents the EDX spectrum of the neat membrane, and it clearly shows no trace of NMO in the composition. The major constituted elements are the C and F, which were the primary elements of the polymeric materials (PVDF/PEG). However, Figure 4b–e has evidenced the presence of the modified Nanocomposite membranes with various loadings of NMO particles. As expected, Mg composition was observed in the spectrum of all the modified fibers at distinct intensities with respect to the NMO dosage. The mapping of Mg in EDX spectra indicates successful blending of the NMO into the matrix structure as well as on the surface of the membranes. Despite the compatibility, only the 0.25 g-NMO and 0.5 g-NMO modified membranes present a free agglomeration with the matrix as depicted in Figure 4b,c. However, at higher NMO loading (0.75 and 1.25 g), an inhomogeneity dispersion and cluster of particles were observed (Figure 4d,e). Reports have shown that presence agglomerated particles in dope solution increases the viscosity, and the this results to irregular nucleation along with uneven crystallization process during the phase separation process [36]. 

#### 3.1.4. Surface Charge of Nano-MgO Membrane

**Figure 5:**
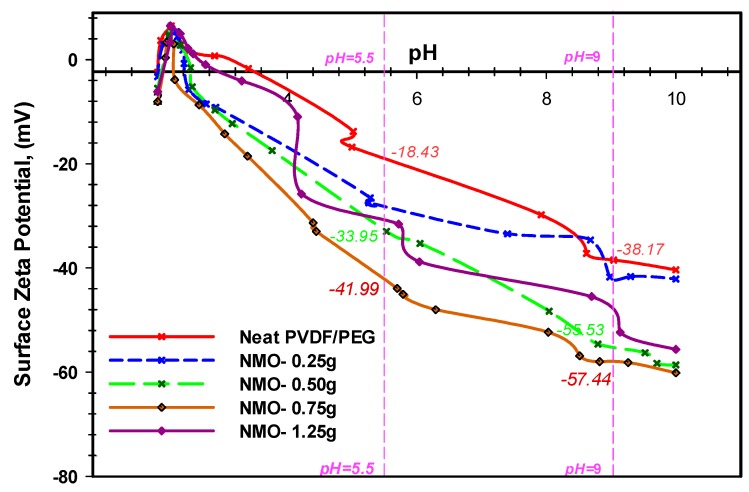
Surface zeta potential of neat PVDF-PEG, and modified Nano-hybrid membranes.

The surface charges of the neat and modified membrane were examined using surface zeta-potential analyzer. The analysis of the surface zeta-potentials for the fabricated hollow fibers with respect to the varied wide range of pH (2 to 10) are presented in Figure 5. The equilibrium isoelectric point of the neat membrane was observed at the pH 3.6, which is in agreement with the previous study [49]. However, the addition of NMO into the dope formulation significantly influenced the surface negativity of the membranes due to the oxidation effect and the formation of the acidic oxides [17]. As shown in Figure 5, the surface of the membranes became more negatively charged with the increasing amount of NMO. This indicates that the NMO became hydrated when added into DMF solvent [17,47]. During the spinning process, the NMO exposed to the membrane surface were hydrolyzed to form some hydroxyl functional groups in the presence of water. The protonation of the NMO has resulted in the deprotonation of membrane surface [42]. Thus, the membrane surface became more negatively charged [17,25]. The fiber with 0.75 g NMO loading presented the highest negative zeta potential within the pH range of 5.5 and 9 with value of −41.99 and −57.44, respectively (Figure 5). The lower surface charges in the 1.25 g NMO loading might be due to excessive agglomeration which resulted in the uneven dispersion and reduction in overall active-surface-area [42]. The membrane with 0.5 g NMO loading recorded higher zeta potential than the fiber with 0.75 g loading, (Figure 5). From same figure, it is obvious that the neat membrane had the least zeta potential at both extreme of the pH range (5.5 and 9) with value of −18.43 and −38.17, respectively. Based on this result, it can be deduced that to derive intensive surface negativity, NMO loading should be maintained between 0.5 and 0.75 g. One of the important applications of negatively surface charged membrane is in the separation of like-charged color pigments; such as the lignin and tannin substances in a typical oily wastewater that contains POME [9]. 

More so, researchers have reported that the main effect of the deprotonation process of a Nano-composite polymeric membrane is the resulted hydroxyl functional groups (OH–) formed on the membrane interacting surface in aqueous media, and this accounted for the surface negatively charge zeta potential [17,25]. More interestingly, the presences of this OH– on the membrane interacting interface improves its water-liking properties (hydrophilicity), permeability as well as repulsion of hydrophobic foulants, such as extracellular polymeric substance (EPS) [9,17]. This remark is strongly in agreement with other studies [17,39,46].

### 3.2. Effect of Nano-MgO on Membrane Performance

#### 3.2.1. Permeability

**Figure 6:**
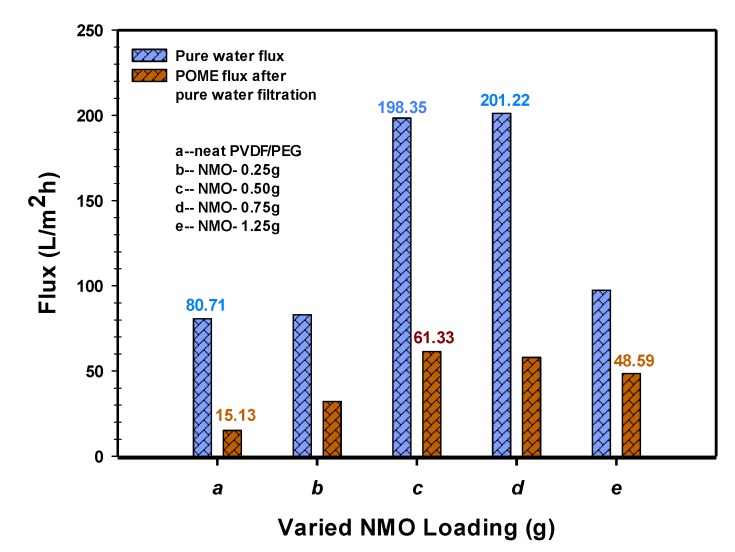
Flux of pure water and POME for neat PVDF-PEG (**a**), and modified Nano-hybrid fibers (**b**–**e**).

Permeability results of the spun fibers both in pure water and diluted POME (4285 ADMI concentration) are shown in Figure 6. The modified fibers demonstrated higher permeate flux performance in both pure water and the diluted-POME. The modified membrane with 0.5 g-NMO and 0.75 g-NMO loading recorded almost equal water flux with 198.35 L/m^2^·h and 201.22 L/m^2^·h, respectively. The 1.25 g-NMO and 0.25 g NMO modified membrane had 97.40 L/m^2^·h and 83.02 L/m^2^·h water flux, respectively, whereas, the neat membrane presented the least flux of 80.72 L/m^2^·h. Based on the flux pattern, it shows that the addition of NMO has a positive impact on the permeability, and this may be due to its ability to enhance the hydrophilicity through the protonation process. However, this effect seems otherwise at higher NMO loading of 1.25 g, Figure 6. This may be attributed to the suppressed pores and dense structure which were the consequential effect of the excessive aggregated particles present within the matrix structure.

Similar trend was observed in the filtration of the diluted-POME. Both 0.5 g-NMO and 0.75 g-NMO modified membranes presents higher flux of 61.33 L/m^2^·h and 57.97 L/m^2^·h, respectively. On the contrary, the neat membrane had the least flux of 15.13 L/m^2^·h, while the 1.25 g-NMO modified membrane recorded 48.59 L/m^2^·h. Collectively, the magnitudes of the fluxes in POME filtration were noticeably lower compared to the pure water filtration. This could be due to the presence of contaminants (such as suspended solids, color pigments, organic and inorganic substances) in the POME, which apparently restrict the free flow of permeate through the membranes. Basically, the selectivity and rejection of the contaminants is based on size differences in the pores as well as the surface zeta potential of the membrane [17]. In addition, the results of the pure water flux after POME filtration indicated that membrane with 0.50 g NMO loading had an outstanding recovery with flux of 183.11 L/m^2^·h.

#### 3.2.2. Rejection of Color Pigment from POME

**Figure 7:**
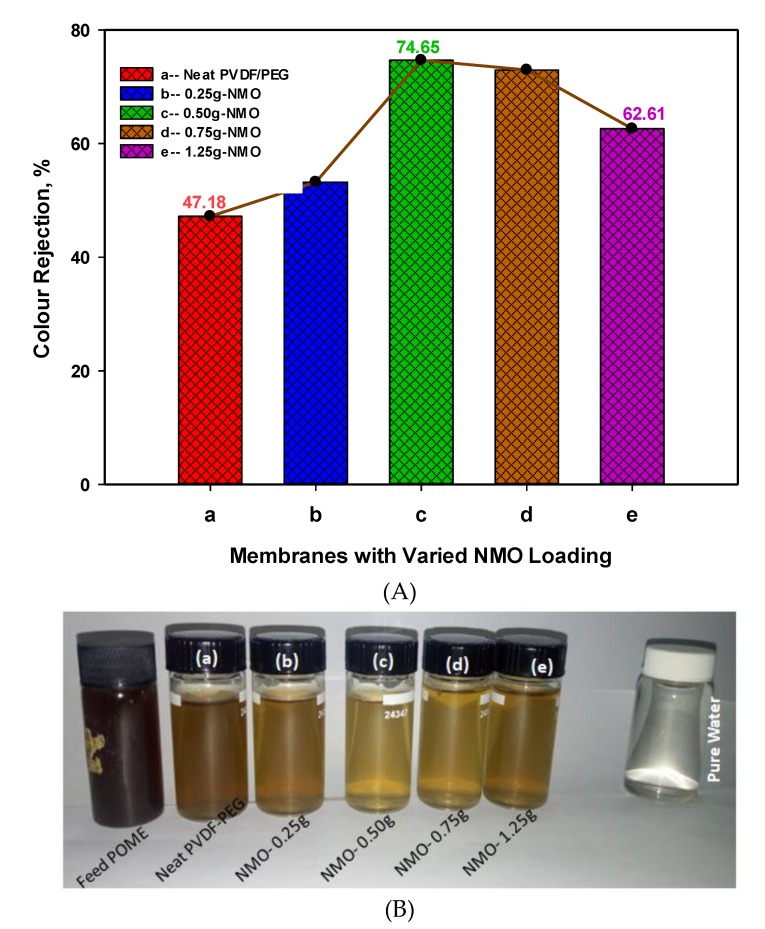
(**A**) Color rejection performance of neat PVDF-PEG (**a**) and modified Nano-hybrid membrane (**b**–**e**). (**B**). Pictorial view of color differences in the feed POME, permeate of (**a**) neat, (**b**–**e**) modified membranes, and pure water.

Figure 7A presents the color removal efficiency for both neat and modified membranes. Noticeably, the membrane with 0.50 g and 0.75 g NMO loading demonstrated outstanding color removal with 74.65% and 72.94%, respectively. It can be noticed that despite the high loading of 1.25 g-NMO, the modified fiber “e” recorded a lower rejection of 47.18% compared to the membrane with 0.50 g-NMO loading. The reason may not be devoid of the inhomogeneity dispersion of the NMO particles which instigated diminution in the effective interacting surface area required to repel the color pigments [36]. Also, Figure 7B display the pictorial visual color differences in feed POME, permeate of the neat and modified membrane as well as pure water. Principally, pore size is a key factor in the separation of color pigment from the POME [17]. This shows that the membrane with pores sizes smaller than the size of the contaminants is capable of giving higher rejection efficiency, though the permeability performance may diminish [50,51]. Besides, the distinct upturn rejection efficiency obtained was not only influenced by the pore sizes but majorly due to the strong negatively charge surface zeta potential. Basically, the surface negativity of the membrane was developed as a result of NMO protonation effect in the presence of water, as explained earlier. In this study, the fed POME has a pH of 8.45 and according to the previous studies, the pH ranging from 8 to 9 was due to the contained color pigments (lignin and tannin) [17,47]. The pigments became negatively charged when the pH of feed was adjusted to acidic conditions (ranging from pH 5.5), this assist in the aggregation of the color pigments [17]. Thus, under this condition of likes negatively charges of color pigments and membrane surface, repulsion of the color pigment prevails during the filtration. This phenomenon considerably improved the color removal efficiency, as observed in the modified fiber (c) and (d).

**Figure 8:**
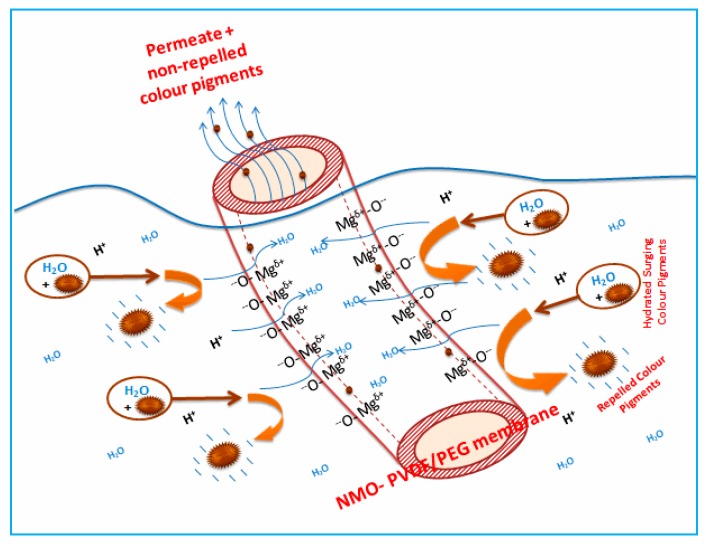
Schematic of color rejection and antifouling mechanism of modified Nanocomposite NMO-PVDF/PEG membranes during filtration.

Figure 8 depicts a simple representation of the color rejection mechanism based on surface negativity of the membrane. As mentioned earlier, the principle of the color rejection was based on the protonation process of both the color pigments present in the diluted POME and NMO to form like charges on the surfaces [47]. During filtration, the hydrated and negatively charged color pigments in the POME migrated towards the surface of the membrane. The presence of water in the medium preceded the protonation of the NMO contained on the surface within the matrix of the membrane, thus resulted in the formation of negative charges. The surging negatively charged pigments were repelled when they approached the membrane surface with like charges [45]. The repulsion of the pigments prevented the deposition of hydrophobic substances (tannin and lignin) on the membrane surface and pore walls. On this basis, the rejection of color pigments not only improves the separation efficiency but also enhance fouling control. Though, some of the un-repelled color pigments with smaller sizes were still able to navigates through the membrane pores along with permeate.

#### 3.2.3. Antifouling Performance and Membrane Reusability

Fouling involves deposition of foulants on the surface and pore walls of a polymeric membrane. Several reports have shown that hydrophobic membrane are more prone to severe fouling due to the strong adhesive attraction that exist between the interacting interface [41,52]. This implies that suppressing the hydrophobic properties through the incorporation of hydrophilic Nanoparticles (NMO) into the membrane matrix structure has the potential to curtail foulants deposition, hence the improvement in antifouling properties. On this note, 3 cycles of POME filtration analysis were conducted in accordance to Subramaniam et al. [9] procedure. Throughout, the filtration time and color concentration of the feed were maintained at 180 min and 4285 ADMI for each of the cycles. At the end of each filtration cycle, the membranes were only physically cleaned under running tap water for 15 min. Essentially, this experiment gives the basis to examine the membrane reusability and antifouling performance using FR, RF and IF as the indicators.

**Figure 9:**
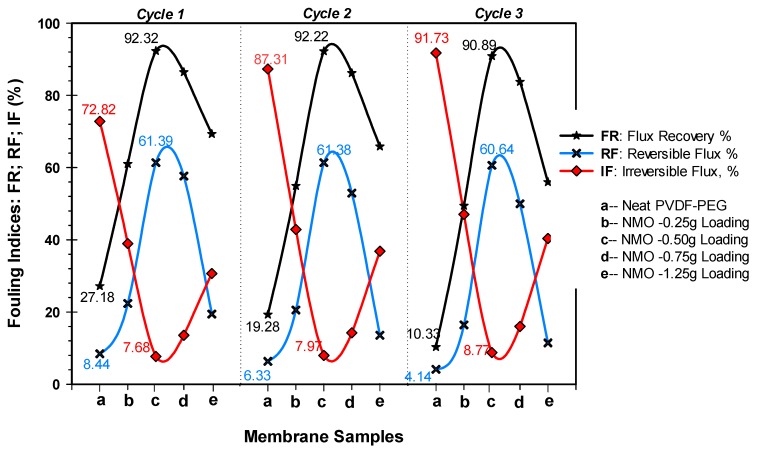
Fouling characteristics (FR, RF and IF) of membrane fibers during three (3) consecutive filtration cycles using POME as feed.

Figure 9 presents all the 3 filtration cycles’ antifouling performance of the spun membranes. From the cycle 1, 0.5 g-NMO modified membrane recorded the highest FR of 92.32%, while the neat membrane had 27.18%. The FR of 1.25 g, 0.75 g and 0.25 g NMO modified membranes were 69.32%, 86.45% and 61.05%, respectively. Furthermore, the fouling resistance performances of the modified membranes were also superior to the neat membrane. The RF% and IF% of the 0.25 g-NMO, 0.5 g-NMO, 0.75 g-NMO and 1.25 g-NMO were (22.36, 38.95), (61.39, 7.68), (57.64, 13.55) and (19.44, 30.68), respectively, as against (8.44, 72.82) for that of the neat membrane. Even at the end of the third filtration cycle 3, the membrane (c) with 0.50 g NMO loading recorded an FR over 90% with good fouling resistance RF% and IF% at 60.64 and 8.77%, respectively. On the contrary, the flux recovery and antifouling resistance of the neat membrane deteriorated significantly with FR%, RF% and IF% of 10.33%, 4.14% and 91.73%, respectively.

Overall, the better antifouling performance of the resultant modified membranes was due to the NMO which essentially improved the hydrophilicity alongside with the negatively surface zeta-potential [25]. Particularly, membrane with 0.50 g loading showed a remarkable improved antifouling performance, and only loss of 1.43% FR with relatively steady RF and IF were observed at the end of the third filtration cycles-3 using diluted-POME as feed. This excellent antifouling performance of the membrane at this loading may be due to the homogenous dispersion of the Nanoparticles, thereby creating superior interacting surface for effective repellence of foulants [36]. More so, apart from the protonation purpose of the NMO in the presence of water to develop a negative charges on the membrane surface, it is also capable of generating free reactive oxygen species (ROS) to inhibit bio-fouling [36]. Thus, preventing the formation of bio-film and cake layer on the membrane surface and pore walls. In addition, Hikku et al. [42] reported that the NMO antifouling activity has a correlation with magnitude of surface area of the Nanoparticle contacting with the microbes (foulants). This implies that evenly dispersed NMO within the membrane matrix presents larger interacting surface area, thus advancing antifouling as well as flux recoverability [25]. 

#### 3.2.4. FTIR Analysis of used Membranes

**Figure 10:**
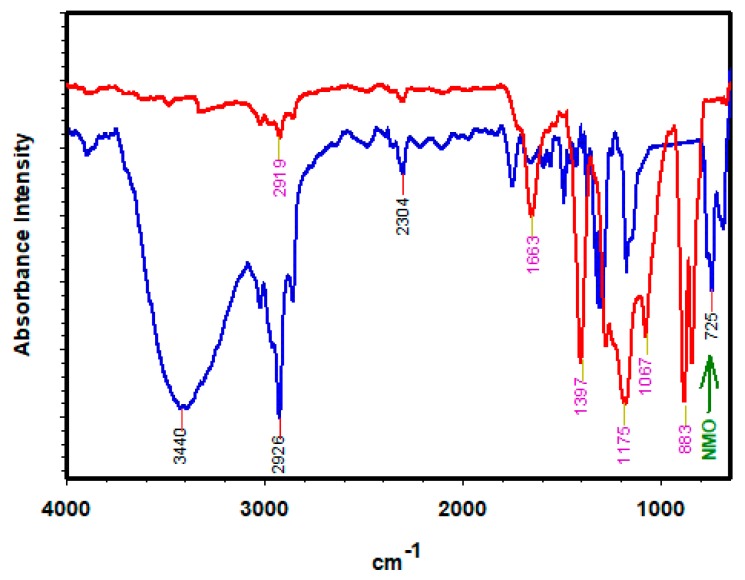
FTIR spectra of used neat membrane (red spectrum), and the modified membrane (blue spectrum).

Figure 10 compares the FTIR spectra of the fouled neat membrane (red spectrum) and the modified membrane (blue spectrum) after-used. The modified-membrane had a broad absorbance at 3440, 2926–2304 and 725 cm^−1^, and these can be attributed to the stretch disturbance of O–H, C–H and Mg–O, respectively [40]. The first functional group (O–H) often indicate the degree of surface hydrophilicity and antifouling properties [24,40,53], as accounted by the broad bands of O–H (blue spectrum). In addition, the FTIR spectra suggest that Nano-MgO (NMO) was successfully incorporated into the PVDF-PEG matrix structure using phase inversion technique. On the contrary, no similar band observed in the neat membrane spectra (red spectrum). The noticeable bands in the neat membrane spectra appeared at 1663, 1397, 1175, 1067 and 883 cm^−1^ which can be attributed to stretch vibrations of amide-I, methyl bonds, CF_2_, polysaccharides and complex aromatic functional group, respectively [54,55]. It was noticed that the stretch band at 1175 cm^−1^ was peculiar in both spectra which resulted due to intrinsic vibration of asymmetric CF_2_ of the PVDF-PEG composition [56]. As suggested by the spectra (red spectrum) of the neat membrane, among the most prevailing functional groups the protein (amide-I 1663 cm^−1^) and polysaccharides (1067 cm^−1^) were both included. These are major source of EPS and SMP substance that are usually responsible for the initiation and formation of biofilm which eventually degenerates into cake layer [57]. Therefore, this shows that the neat membrane is highly prone to fouling. On the contrary, the deprotonating process of the Nano-MgO (NMO) improves the intensity of the reactive O-H (blue spectrum) in the modified membrane. Thus, generated O–H denaturize the deposited EPS/SMP as well as improving the hydrophilicity of the polymeric membrane [40]. This phenomenon effectively averts formation of the cake layer on the modified membrane, as observed in Figure 10.

### 3.3. Performance Appraisal with Literatures

This section presents a concise comparison of this study with previous works that uses MgO Nanoparticle (NMO) to modified polymeric membranes. In this study, NMO was successfully incorporated into the PVDF-PEG matrix using phase inversion technique. This resulted in considerable augmentation of the hydrophilicity and surface negativity of the spun hollow fibers. At 0.50 g NMO loading, a remarkable water flux, and POME permeability flux along with the color rejection of 198.35 L/m^2^·h, 61.33 L/m^2^·h and 74.65% were obtained, respectively. Even after the 3 filtration cycle of a total period of 9 h, the flux recovery percentage remains relatively steady at 92.32%. Previously, Parvizian et al. [39] reported that at a varied loading of NMO blended into PVC matrix, the flux improves and the highest flux was obtained at 0.5 wt % dosage. However, a comprehensive fouling analysis and color rejection of the PVC membrane were not considered. Arumugham et al. [58] used NMO to modify sulfonated polyphenyl sulfone (SPPSU) polymeric membrane and applied to treat oily wastewater. The NMO loading was fixed at 25 wt % while the SPPSU and organic solvent were varied. From the results, a good hydrophilic performance (99.00% of flux recovery) was achieved. However, the improvement in the hydrophilicity may not be attributed to incorporated NMO since the dosage was not varied [58]. Based on this appraisal, it can be deduced that this study not only bridge the information gap, but also reported the significant role of the NMO at varied dosage in improving performance of the hybrid PVDF-PEG membrane.

## 4. Conclusions

Hybrid PVDF-PEG hollow fibers blended with NMO at various loading of 0–1.25 g were fabricated using phase inversion technique. The increasing NMO loading has demonstrated a significant effect on the morphology, hydrophilicity, permeability and antifouling properties of the resultants composite hollow fibers. The loading at 0.50 g-NMO presented the most auspicious performance with 198.35 and 61.33 L/m^2^·h of pure water and POME filtration flux, respectively. After a continues 3 filtration cycles of diluted-POME for a total period of 9 h, the 0.50 g-NMO composite membrane recorded the best flux recovery (FR), reversible fouling (RF) and least irreversible fouling (IF) percentages of 90.98%, 61.39% and 7.68%, respectively. The outstanding performance was due to the homogenous distribution and compatibility of the membrane matrix with the NMO particles at 0.50 g loading. Conversely, after the third filtration cycle-3, a significant deterioration in the FR and fouling resistance (RF and IF) were noticed at higher loading of 1.25 g-NMO with a values of 55.98%, 11.44% and 40.38%, respectively. This was due to the excessive NMO loading which resulted in the formation of numerous aggregated particles within the matrix structure. The agglomerated particles skewed the nucleation and protonation process during the phase separation and crystallization, as well as creating weak spots in the resultant membranes. Therefore, it can be deduced that Nano-hybrid PVDF-PEG membrane with 0.50 g-NMO loading presents the best performance. Overall, the modified fibers presented better performances compared to the neat membrane.

## 5. Patents

Malaysian Patent No. PI 2019006570, 2019: A Hybrid System and Method for Treating Palm Oil Mill Effluent.

## Figures and Tables

**Table 1:** Chemical formulation of Neat PVDF-PEG and modified Nano-hybrid membranes.

Samples	PVDF/PEG (g)		DMF (g)	LiCl.H_2_O (g)	N*MO* (g)	Mass of Dope (g)
**Neat PVDF/PEG**	30/10	(3:1)	158	2.0	--	200.00
**NMO-0.25 g**	30/10	(3:1)	158	2.0	0.25	200.25
**NMO-0.50 g**	30/10	(3:1)	158	2.0	0.50	200.50
**NMO-0.75 g**	30/10	(3:1)	158	2.0	0.75	200.75
**NMO-1.25 g**	30/10	(3:1)	158	2.0	1.25	201.25
